# Adams–Oliver Syndrome: A Comprehensive Literature Review of Clinical, Nutritional, Genetic, and Molecular Aspects with Nursing Care Considerations

**DOI:** 10.3390/ijms27010173

**Published:** 2025-12-23

**Authors:** Ioana Badiu Tișa, Anamaria Cozma-Petruț, Alin-Dan Chiorean, Doina Miere, Lorena Filip, Roxana Banc, Oana Mîrza, Mădălina Adriana Bordea

**Affiliations:** 1 Department 1, Faculty of Nursing and Health Sciences, “Iuliu Hațieganu” University of Medicine and Pharmacy, 2-4 Câmpeni Street, 400217 Cluj-Napoca, Romania; ioana.badiu@umfcluj.ro; 2 Department of Bromatology, Hygiene, Nutrition, Faculty of Pharmacy, “Iuliu Hațieganu” University of Medicine and Pharmacy, 6 Pasteur Street, 400349 Cluj-Napoca, Romania; dmiere@umfcluj.ro (D.M.); lfilip@umfcluj.ro (L.F.); roxana.banc@umfcluj.ro (R.B.); oana.stanciu@umfcluj.ro (O.M.); 3 Department of Cell and Molecular Biology, Faculty of Medicine, “Iuliu Hațieganu” University of Medicine and Pharmacy, 6 Pasteur Street, 400349 Cluj-Napoca, Romania; chiorean.alin@umfcluj.ro; 4 Academy of Romanian Scientists (AOSR), 3 Ilfov Street, 050044 Bucharest, Romania; 5 Department of Microbiology, Faculty of Medicine, “Iuliu Hațieganu” University of Medicine and Pharmacy, 6 Pasteur Street, 400349 Cluj-Napoca, Romania; madalina.bordea@umfcluj.ro

**Keywords:** Adams–Oliver syndrome, genetics, molecular mechanisms, limb anomalies, skin defects, heart defects, brain anomalies, nutritional issues, nursing care

## Abstract

The present review aims to provide a comprehensive overview of the current literature on Adams–Oliver syndrome (AOS), synthesizing information on its clinical features, genetic and molecular underpinnings, nutritional aspects, and key nursing care considerations. AOS is a rare congenital disorder. Its genetic basis is heterogeneous, involving mutations in at least six key genes (*ARHGAP31*, *RBPJ*, *NOTCH1*, *DLL4*, *DOCK6*, and *EOGT*), which primarily affect vascular development through pathways like Notch signaling and Rho GTPase regulation. The management of AOS is complex and requires a multidisciplinary approach. The clinical presentation of AOS is highly variable, ranging from mild to severe and includes a wide spectrum of clinical manifestations, most notably aplasia cutis congenita and terminal transverse limb defects. The underlying molecular mechanisms predominantly point towards vasculopathy, disrupting embryonic development. Emerging evidence also highlights the presence of nutritional issues, such as poor feeding and growth failure, which are often overlooked. Management demands an integrated, multidisciplinary management approach, requiring coordinated effort from specialists in pediatrics, genetics, molecular biology, cardiology, surgery, and nutrition. Specialized nursing care is crucial for managing complex symptoms, particularly wound care for aplasia cutis, and for providing family support.

## 1. Introduction

Adams–Oliver syndrome (AOS) was initially described by Forrest Adams and Clarence Oliver in 1945 [1,2], and since then, several similar cases have been reported worldwide [3,4,5,6]. The prevalence is unknown, but it is estimated to affect approximately 1 in 225,000 live births [7]. AOS has been reported in populations worldwide, but the current body of literature suggests no clear racial or ethnic predilection. While case reports originate from numerous countries, a lack of systematic reporting of ethnicity prevents a quantitative analysis of its distribution among different ethnic groups [8].

The clinical spectrum of AOS is broad, with variable expressivity and genetic heterogeneity. The genetic basis of AOS is complex, with mutations in at least six genes (*ARHGAP31*, *RBPJ*, *NOTCH1*, *DLL4*, *DOCK6*, and *EOGT*) identified as causative [9]. Given the rarity of the syndrome and its variable presentation, diagnosis and management pose significant challenges, requiring a multidisciplinary approach. The present review aims to provide a comprehensive overview of Adams–Oliver syndrome. The review explores the clinical features, genetic underpinnings, and molecular mechanisms of the syndrome and discusses the importance of an integrative approach to patient care, targeting clinical, nutritional, genetic, and molecular aspects.

## 2. Methods

The present manuscript is a comprehensive narrative review. A literature search was conducted using the PubMed and Google Scholar electronic databases to assess the information published on Adams–Oliver syndrome. Moreover, websites of organizations in the rare disorders field were accessed to find additional information.

The literature search covered studies published between 1995 and 2025. The search strategy utilized a combination of Medical Subject Headings (MeSH) and free-text keywords. The primary search terms included: “Adams-Oliver Syndrome” OR “AOS”, “Aplasia Cutis Congenita” AND “Limb Defects”, “Terminal Transverse Limb Defects”, the specific genes associated with AOS (“ARHGAP31”, “DOCK6”, “RBPJ”, “NOTCH1”, “DLL4”, “EOGT”), and combined searches such as (“Adams-Oliver Syndrome”) AND (“Nutrition” OR “Feeding” OR “Growth”) and (“Adams-Oliver Syndrome”) AND (“Nursing” OR “Wound Care”).

Articles were selected for inclusion if they met the following criteria: (1) were original research articles, case reports, case series, or review articles; (2) focused on the genetic, molecular, clinical, nutritional, or nursing aspects of AOS; and (3) were published in the English language. Articles were excluded if they were not in the English language or if they were not relevant to the topic of the current review.

The selection of articles aimed to build a comprehensive overview of the current state of knowledge. Nevertheless, several inherent limitations associated with the review methodology should be noted. As a narrative review, the study selection may be susceptible to selection bias. Furthermore, the literature on rare diseases like AOS is prone to publication bias, where studies reporting positive or more severe outcomes are more likely to be published than those reporting mild or negative findings [10]. This can lead to an overestimation of the frequency and severity of certain clinical features. To address this limitation, a broad range of case reports and series were included. 

## 3. Genetic and Molecular Basis of Adams–Oliver Syndrome

AOS is counted among rare congenital diseases. The occurrence of the disease is mostly familial. Nevertheless, a subset of cases can manifest as isolated, sporadic occurrences. AOS exhibits considerable clinical variability, from mild to severe phenotypes, with a wide array of features, particularly aplasia cutis congenita and terminal transverse limb defects. The pathophysiology of AOS is incompletely understood, with multiple mechanisms warranting in-depth investigation. Many theories primarily focus on vascular pathogenesis, such as congenital small vessel defects, impaired pericyte recruitment to vessels, or disruption of early embryonic circulation in affected vascular areas. Therefore, AOS is considered a genetic defect that causes vasculopathy and leads to various phenotypes involving the lungs, brain, liver, kidneys, eyes, and skin [3,11,12,13,14,15,16].

### 3.1. Overview of AOS-Associated Genes and Inheritance Patterns

AOS is caused by genetic mutations in at least six key genes, including *ARHGAP31*, *RBPJ*, *NOTCH1*, *DLL4*, *DOCK6*, and *EOGT* [17,18,19,20]. Depending on the gene involved, AOS exhibits both autosomal dominant (AD) and autosomal recessive (AR) inheritance patterns [9,11,21,22,23,24,25].

Autosomal dominant forms of AOS are linked to *ARHGAP31* variants, while *DOCK6* or *EOGT* variants are associated with a recessive inheritance pattern [13]. Additional autosomal dominant variants of Adams–Oliver syndrome include AOS3, AOS5, and AOS6. A mutation in the *RBPJ* gene located on chromosome 4p15 results in the AOS3 form. As for the AOS5 form, it is determined by a mutation in the *NOTCH1* gene situated on chromosome 9q34, whereas the AOS6 form is caused by a mutation in the *DLL4* gene located on chromosome 15q15.1. Furthermore, autosomal recessive forms of Adams–Oliver syndrome comprise AOS2, determined by a mutation in the *DOCK6* gene situated on chromosome 19p13, and AOS4, resulting from a mutation in the *EOGT* gene located on chromosome 3p14 [26,27,28,29,30,31,32].

Genetic testing can be used to identify mutations in any of the established genes linked to AOS, but this has been found to account for roughly 30% of the cases with this syndrome to date. As a result, not every case can benefit a complete diagnosis [13,30,33,34,35,36].

### 3.2. Molecular Functions of Key AOS-Related Proteins

The specific molecular mechanisms by which the causal AOS genes influence its pathogenesis are presented in this section, as well as summarized in Table 1.

#### 3.2.1. ARHGAP31

*ARHGAP31* (Rho GTPase Activating Protein 31) on chromosome 3q13 is a gene that encodes the ARHGAP31 protein, a GTPase-activating protein (GAP), which exhibits essential functions in the division, survival, and migration of cells [17,40]. The primary role of GAPs is to inactivate small Rho GTPases such as Cdc42 and Rac1 by stimulating the hydrolysis of their bound GTP to GDP. Thus, ARHGAP31 acts as a negative regulator of the signaling mediated by Cdc42 and Rac1, which are important molecular switches for a multitude of cellular processes. For instance, Cdc42 and Rac1 play a key role in actin cytoskeleton organization, controlling actin dynamics, which is essential for cell shape, movement, and polarity. Likewise, Cdc42 and Rac1 are fundamental for cell migration, a vital process during embryonic development and angiogenesis, as well as for cell adhesion (cell–cell and cell–extracellular matrix interactions) and vascular development [17,37,41].

The mutations in the *ARHGAP31* gene associated with the AOS1 dominant form are often gain-of-function mutations, conducting to the synthesis of ARHGAP31 proteins with increased GAP activity, further leading to excessive and constitutive inactivation of small GTPases Cdc42 and Rac1. This disruption of the small GTPase signaling balance has severe consequences for cellular processes dependent on them, especially during embryonic development. Constant inactivation of Cdc42 and Rac1 disrupts normal cytoskeleton organization and it also alters cell migration, a process that is critical for the proper development of tissues, including blood vessels and limbs. Furthermore, excessive inactivation of Cdc42 and Rac1 leads to altered angiogenesis and vasculopathy, which manifests as defects in blood vessel formation. This is considered the fundamental cause of cutaneous malformations (aplasia cutis congenita) and limb defects observed in AOS [17,37,41].

Indeed, the mutations in the *ARHGAP31* gene lead to classic AOS features, including scalp defects and limb abnormalities. Cutis marmorata telangiectatica congenita is also common in AOS1. Southgate et al. reported cases of AOS1 associated with a gain-of-function mutations in the *ARHGAP31* gene and presenting with aplasia cutis congenita of the scalp and terminal transverse limb defects affecting both upper and lower extremities [17]. Furthermore, Santaniello et al. described another case of AOS1, involving a 33-year-old woman with hypoplastic lower extremity fingers in both feet, dimensional reduction in all proximal phalanges, and absence of all distal phalanges. Clinical examination revealed mild thinning of the cutis at the vertex region without alopecia. Clinical exome sequencing identified a novel heterozygous frameshift variant (c.2193del, p.Thr732Glnfs*26) in exon 12 of the *ARHGAP31* gene. The variant segregated in an autosomal dominant pattern, with the proband’s brother showing toe shortening and bilateral partial syndactyly, and the father exhibiting partial lower extremity digit syndactyly, demonstrating variable expressivity [40].

#### 3.2.2. RBPJ

The protein encoded by *RBPJ* (recombination signal binding protein for immunoglobulin kappa J region) gene is a major transcription factor that plays a central role in the Notch signaling pathway [38,42]. The Notch pathway is essential for intercellular communication during embryonic development, controlling critical processes such as cell proliferation, differentiation, and apoptosis [20].

In the absence of a Notch signal, RBPJ binds to DNA in the promoter regions of Notch target genes and acts as a transcriptional repressor, recruiting co-repressors to block gene expression. When a cell receives a Notch signal through the interaction between a Notch ligand (e.g., Delta or Jagged ligand) and a Notch receptor, the intracellular portion of the receptor (the Notch intracellular domain, NICD) is released, translocates to the nucleus, and binds to RBPJ. This NICD-RBPJ complex recruits transcriptional co-activators, transforming RBPJ from a repressor into a transcriptional activator and initiating the expression of Notch target genes [43,44].

Mutations in the *RBPJ* gene associated with dominant forms of AOS lead to haploinsufficiency or altered function of the RBPJ protein. These mutations often affect the DNA-binding domain of the RBPJ protein, with two molecular consequences. One is a compromised DNA binding, with the mutant RBPJ protein not being able to efficiently bind to the regulatory regions of Notch target genes. This prevents both basal repression (in the absence of a signal) and proper activation (in the presence of a signal) of these genes [20,38]. The second molecular consequence is a dysregulation of Notch target gene expression, which results in a dysregulation of the Notch signaling pathway. Because this pathway is essential for vascular development, its disruption leads to vasculopathy and defective angiogenesis. The resulting vascular defects explain both the aplasia cutis congenita that is caused by insufficient vascularization of the skin during fetal development and the terminal limb anomalies, where the proper development of bones and soft tissues is dependent on an adequate vascular network [20,29,38].

Hassed et al. reported the cases of two independent families with autosomal dominant AOS associated with heterozygous missense mutations in the *RBPJ* gene. In family 1, the proband presented at birth with cutis aplasia of the scalp, syndactyly of the second and third toes, microcephaly, and mild motor delay, while her father exhibited short distal phalanges of the fingers, absent toes with short metatarsals, microcephaly, and intellectual deficits. Molecular analysis revealed a heterozygous mutation (c.188A>G, p.Glu63Gly) in the DNA-binding domain of the *RBPJ* gene. In family 2, affected individuals showed variable phenotypes ranging from mild limb reductions to severe manifestations including large scalp scars from cutis aplasia, asymmetric hand and foot reductions, and intellectual deficits. A distinct heterozygous mutation (c.505A>G, p.Lys169Glu) was identified in the *RBPJ* gene. Functional assays confirmed that both mutations significantly impaired RBPJ DNA-binding affinity [20].

#### 3.2.3. NOTCH1

The *NOTCH1* gene encodes the Notch1 protein, a transmembrane receptor that plays a crucial role in cell-to-cell communication through the Notch signaling pathway [25]. The NOTCH1 protein has a complex structure, including an extracellular domain with multiple epidermal growth factor (EGF)-like repeats, which are essential for ligand binding, and an intracellular domain (NICD) that acts as a transcriptional regulator [28,39].

As previously mentioned, the Notch signaling pathway is activated when a ligand from a neighboring cell (such as Delta-like ligands DLL1, DLL3, DLL4 or Jagged ligands JAG1, JAG2) binds to the extracellular domain of the NOTCH1 receptor. This interaction triggers a series of proteolytic cleavages of the NOTCH1 protein. The final cleavage, mediated by the γ-secretase complex, releases NICD. The NICD then translocates to the nucleus, where it forms a complex with the DNA-binding protein RBPJ and co-activators of the Mastermind-like (MAML) family. This complex activates the transcription of target genes that regulate cell fate decisions (e.g., proliferation, differentiation, apoptosis) [28,45].

Mutations in the *NOTCH1* gene have been identified as a primary cause of AOS. These mutations can disrupt the normal function of the Notch signaling pathway in several ways, leading to the clinical features of AOS. The primary mechanism is often haploinsufficiency, where one of the two copies of the *NOTCH1* gene is inactivated, resulting in an insufficient amount of the NOTCH1 protein required for normal function. This can be caused by large deletions of the gene or by mutations that introduce a premature stop codon, affecting the function of the protein [28].

Specific mutations in the *NOTCH1* gene can have distinct effects on the NOTCH1 protein’s function. For instance, splice-site mutations can lead to the exclusion of essential exons, affecting the structure of the EGF-like repeats in the extracellular domain. This can impair ligand binding and, consequently, the activation of the Notch pathway. Furthermore, missense mutations, resulting in the substitution of a single amino acid, can also have severe consequences. For example, mutations affecting cysteine residues can disrupt disulfide bridges that are critical for the proper folding and stability of the protein. This can lead to a destabilized protein that is unable to function correctly. Disruption of the Notch signaling pathway due to *NOTCH1* mutations is thought to particularly affect the development of the vascular system further leading to tissue damage and the developmental abnormalities observed in AOS [28].

Furthermore, when discussing the molecular basis of AOS, it is worth discussing the potential link between NOTCH1 signaling and mitochondrial metabolism. While mitochondrial dysfunction is not considered a primary pathogenic mechanism in AOS, an emerging body of evidence reveals an indirect link through the dysregulation of the NOTCH1 signaling pathway, which is a known regulator of cellular energy metabolism. Studies have demonstrated that aberrant NOTCH1 signaling can alter the mitochondrial proteome and directly impact mitochondrial function [46,47]. Therefore, it is plausible that the haploinsufficiency of *NOTCH1* in AOS could lead to secondary bioenergetic deficits. Such effects might contribute to the severity of the clinical phenotype, particularly in tissues with high metabolic demand like the developing heart and brain, which are frequently affected in the syndrome. This potential connection, while not fully elucidated in AOS, warrants further investigation.

Indeed, a study by Southgate et al. identified heterozygous loss-of-function mutations in the *NOTCH1* gene as a significant cause of AOS, particularly in cases with cardiovascular anomalies. In two families with autosomal dominant AOS, novel truncating mutations (c.1649dupA and c.6049_6050delTC) were identified. Screening a larger cohort revealed eight additional unique *NOTCH1* mutations. A key finding was the high prevalence of congenital heart defects, present in 47% of individuals with *NOTCH1* mutations, including aortic stenosis, aortic regurgitation, bicuspid aortic valve, and coarctation of the aorta. The study demonstrated that these mutations lead to reduced *NOTCH1* expression and downregulation of Notch target genes (*HEY1*, *HES1*), indicating that *NOTCH1* haploinsufficiency is the underlying molecular mechanism. This established a strong genotype–phenotype correlation, linking *NOTCH1* mutations in AOS to a significantly increased risk of variable cardiac anomalies [29].

#### 3.2.4. DLL4

The protein encoded by the *DLL4* gene, known as Delta-like ligand 4, is a critical component of the Notch signaling pathway [24,48]. At the molecular level, DLL4 functions as a transmembrane ligand that binds to and activates Notch receptors on adjacent cells. As described earlier, this interaction is essential for regulating cell fate decisions, particularly during vascular development. DLL4 is highly expressed in arterial endothelial cells and plays a key role in angiogenesis. It acts as a negative regulator of angiogenic sprouting, ensuring the proper patterning and maturation of the vasculature [34].

A mutation in the *DLL4* gene, specifically a heterozygous loss-of-function mutation, impairs the ability of the DLL4 protein to bind to and activate Notch receptors effectively. This disruption of the DLL4-Notch signaling pathway can lead to disorganized and defective blood vessel formation, conducting to the congenital scalp and limb defects characteristic of AOS [34].

Meester et al. identified heterozygous loss-of-function mutations in the *DLL4* gene as a cause of autosomal dominant AOS. Using a candidate-gene approach in 91 families, nine different heterozygous mutations were identified, including two nonsense (p.Gln554* and p.Arg558*) and seven missense variants. These mutations were found in individuals presenting with a wide spectrum of clinical features, including aplasia cutis congenita, terminal limb defects, and, in some cases, cardiovascular anomalies such as ventricular septal defect and tricuspid insufficiency. The study highlighted the highly variable clinical expression among affected individuals, even within the same family, with no clear genotype–phenotype correlation emerging. These findings established *DLL4* as a causative gene for AOS, further underscoring the crucial role of Notch signaling in the etiology of this disorder [34].

#### 3.2.5. EOGT

The protein encoded by the *EOGT* gene is the EGF domain-specific O-linked N-acetylglucosamine (O-GlcNAc) transferase. At a molecular level, this enzyme resides in the endoplasmic reticulum and catalyzes a crucial post-translational modification known as O-GlcNAcylation. Specifically, it attaches an N-acetylglucosamine sugar molecule to serine or threonine residues within the EGF-like domains of a select group of secreted and membrane-bound proteins. One of the most critical targets for EOGT-mediated glycosylation is the NOTCH1 receptor [19].

An autosomal recessive, loss-of-function mutation in the *EOGT* gene disrupts the enzymatic activity of EOGT, leading to the phenotype of AOS. In the absence of a functional EOGT enzyme, its target proteins, including NOTCH1, are not properly glycosylated. Research has shown that O-GlcNAcylation of the NOTCH1 receptor’s EGF repeats is essential for its effective interaction with its ligands, particularly Delta-like ligands (e.g., DLL4). Therefore, a mutation in EOGT results in impaired binding of these ligands to the NOTCH1 receptor. This failure to activate the Notch signaling cascade correctly is accompanied by consequences, as indicated previously. It disrupts critical developmental processes, most notably vascular development, with the resulting vascular insufficiency being considered an underlying cause of the characteristic congenital defects of AOS (e.g., aplasia cutis congenita, terminal limb abnormalities) [19].

The study conducted by Shaheen et al. was the first to link the *EOGT* gene to the pathogenesis of AOS. Shaheen et al. identified three different homozygous mutations in the *EOGT* gene in three consanguineous families. These included two missense mutations (c.620G>C and c.878G>A) and one frameshift deletion (c.832–791delA). The affected individuals presented with classic AOS features, including aplasia cutis congenita and terminal transverse limb defects, as well as cardiac and neurological abnormalities in some cases [19]. Subsequently, Schröder et al. described two families with *EOGT*-associated AOS, identifying two novel mutations (c.404G>A and c.311+1G>T). The phenotype in these families was characterized primarily by large aplasia cutis congenita, while limb defects were subtle or absent, and no major malformations were observed. One family exhibited a pseudodominant inheritance pattern, which initially complicated the recognition of the recessive mode of transmission. These cases expanded the mutational spectrum of *EOGT*-related AOS and suggested that the phenotype might differ from *DOCK6*-associated recessive AOS, with a lower frequency of neurological or ocular deficits [9].

#### 3.2.6. DOCK6

According to research, *DOCK6* gene is the second causative gene for Adams–Oliver syndrome [49]. *DOCK6* (dedicator of cytokinesis 6) gene (also known as *ZIR1* gene) encodes DOCK6 protein, a member of the subfamily C of the dedicator of cytokinesis (DOCK) family [18]. The specific molecular mechanisms by which DOCK6 and other relevant DOCK proteins in the DOCK family influence vascular development and disease are further detailed.

The primary focus of research on the DOCK family up until recently was on neural development and neurological disorders. Nevertheless, studies conducted during the last decade have shown that DOCK proteins have roles that are more complex when it comes to human illnesses. For instance, it has been shown that DOCK proteins control vascular processes and osteogenesis [49].

The first protein in the DOCK family to be characterized was DOCK1, also known as DOCK180 because it is a protein with a mass of 180 kDa. DOCK1 protein is expressed in every tissue. However, DOCK1 protein expression is notably enhanced in pulmonary artery, human umbilical vein, and microvascular endothelial cells. Research conducted in animals suggested that DOCK1 has a particular role in skeletal muscle development. Unlike DOCK1, which is expressed in every tissue type, DOCK2 expression is mostly seen in hematopoietic cells [49].

When it comes to vascular disease, recent research has linked DOCK3 and DOCK4 to mechanisms for atherogenesis process and neovascularization after an ischemic event. Moreover, DOCK4 and DOCK5 are known to play an essential role in the onset of myocardial hypertrophy and fibrotic changes. Additionally, they are expressed on the stereocilia found in the cochlea and the retinal structures [49].

DOCK6 functions at the molecular level as an atypical guanine exchange factor (GEF) that is vital for the activation of the small GTPases Cdc42 and Rac1. DOCK6 accomplishes the activation by catalyzing the exchange of GDP for GTP on these proteins, thereby switching them to their active, signal-transducing state. As previously noted, Cdc42 and Rac1 play essential roles in regulating cytoskeletal dynamics, cell migration, and angiogenesis, processes that are crucial for normal cellular function and development [18].

Nucleotide substitutions in the *DOCK6* gene, especially point mutations such as single nucleotide changes, insertions, or deletions, critically impair the function of the DOCK6 encoded protein. Mutations often affect the GEF domain of *DOCK6*, disrupting its ability to activate the small GTPases Cdc42 and Rac1, leading to abnormal signaling pathways that impair cell morphology, stability, and vascular development. Specifically, the loss of GEF activity due to these mutations hinders DOCK6’s capacity to facilitate the exchange of GDP for GTP on Cdc42 and Rac1, resulting in deficient signal transduction necessary for cytoskeletal organization and cell movement. Furthermore, some mutations induce structural instability in the DOCK6 protein, making it prone to degradation, which decreases the levels of functional protein available in the cell. These disruptions lead to a cascade of cellular dysfunctions, including impaired cell migration and abnormal angiogenesis, particularly in the limb buds and scalp ectoderm, leading to the characteristic limb and scalp defects observed in AOS [18,50].

The autosomal recessive form of AOS caused by *DOCK6* mutations is characterized by a particularly severe phenotype. In addition to the classic features of aplasia cutis congenita and limb defects, affected individuals frequently present with central nervous system abnormalities and ocular manifestations [51]. Indeed, a case of type 2 autosomal recessive AOS associated with heterozygous mutations in the *DOCK6* gene was reported by Jones et al., showing characteristic findings of aplasia cutis congenita, terminal transverse limb defects, intracerebral periventricular calcifications, and polymicrogyria [52]. Another case of type 2 AOS was presented by Alzahem et al. involving a 4-month-old boy with microcephaly, global developmental delay, truncal hypotonia, and limb reduction defects. Ophthalmic examination revealed bilateral nystagmus, retinal detachment, and a retrolental plaque in the left eye. Next-generation sequencing identified a novel homozygous likely pathogenic variant (c.1269_1285dup (p.Arg429Glnfs*32)) in the *DOCK6* gene, confirming the diagnosis of type 2 AOS [31].

Lehman et al. identified a child with mild aplasia cutis congenita, terminal transverse limb defects, developmental delay, and diffuse angiopathy with incomplete microvascularization. Whole-genome sequencing revealed two rare truncating variants in *DOCK6* [33]. Another patient with autosomal recessive AOS was reported by Shaheen et al., where autozygome analysis and exome sequencing identified a homozygous truncating mutation in the *DOCK6* gene. A cellular phenotype characteristic of defective actin cytoskeleton was observed in the patient’s cells [18].

It can be noted that the genetic heterogeneity of AOS is paralleled by its broad clinical variability. To synthesize the information on genotype–phenotype correlations in AOS, Table 2 provides a comprehensive summary of the major clinical features associated with mutations in the six known causative genes. Nevertheless, genotype–phenotype correlations in AOS must be interpreted with caution. Much of the available data is derived from small family cohorts and individual case reports, and significant phenotypic variability exists even among individuals with the same mutation. For instance, while *NOTCH1* mutations are strongly associated with cardiac defects, not all individuals with these mutations present with cardiac anomalies. A comprehensive review of 398 individuals with AOS found that while certain trends exist, the predictive power of genotype for a specific phenotype remains limited due to the syndrome’s heterogeneity [53].

## 4. Adams–Oliver Syndrome: Clinical Presentation and Diagnostic Criteria

AOS is characterized by abnormal skin development, particularly on the scalp, and various limb malformations. AOS is also known as congenital aplasia cutis associated with terminal transverse limb defects, or as congenital scalp defects with distal limb reduction. Symptoms frequently manifest as areas with absent skin on the scalp that are scarred and hairless. In some cases, the bones beneath the skin are also underdeveloped. Conditions that disrupt the integrity of the skeletal system can typically increase the risk of bleeding. These lesions can become infected in certain cases [1,3,9,53,56,57].

Further manifestations of AOS include heart defects, more commonly dilated veins of the scalp, pulmonary hypertension, brain abnormalities, developmental disabilities, cleft lip, glaucoma, poor feeding, and reduced weight gain [5,6,58,59].

Major features of AOS include terminal transverse limb defects, congenital cutis aplasia, and a family history of AOS. In contrast, minor features comprise cutis marmorata, congenital cardiac defects, and vascular anomalies [60]. It is sufficient to identify either two major features or one major and one minor feature to establish a diagnosis of AOS [4].

### 4.1. Limb Anomalies

Limb malformations in AOS may include nail abnormalities and anomalies of the digits. Suarez et al. indicated that syndactyly, brachydactyly, polydactyly, and oligodactyly are among the most frequently occurring limb defects in AOS [3]. The severity of limb defects varies widely, from complete limb absence to only mild abnormalities. Terminal transverse limb defects involve significant impairment of the distal phalanges or entire fingers. The defects can be observed in both upper and lower limbs, but defects of the lower limbs occur more frequently. Brachydactyly and syndactyly of toes two and three are most frequently reported, though polydactyly and oligodactyly also occur. The nails may appear small, thin, discolored or abnormally shaped. In severe cases, necrosis or digital loss has been reported [8,51,59].

### 4.2. Skin Anomalies

Other associated anomalies reported in AOS include cutis marmorata telangiectatica congenita, characterized by discolored skin patches caused by dilated surface blood vessels [3,4,11,26,52,61]. The cutis marmorata telangiectatica congenita lesions in AOS may present with ulceration, telangiectasia and areas of hypo- or hyperpigmentation, with prominent veins frequently observed on the forehead or scalp [8,51,59].

### 4.3. Heart Anomalies

The involvement of congenital heart anomalies is a frequent manifestation observed in AOS [29]. For instance, Badiu Tișa et al. reported the case of a patient presenting patent foramen ovale as a minor criterion [62]. Zapata et al. mentioned in their study that all patients with AOS should be evaluated for cardiac anomalies [15]. Indeed, it is estimated that congenital heart defects occur in over 20% of individuals with AOS. These include: atrial septal defects (ASD), ventricular septal defects (VSD), patent ductus arteriosus (PDA), coarctation of the aorta (CoA), aortic stenosis, hypoplastic left heart syndrome, bicuspid aortic valve, and pulmonary stenosis. More severe defects such as right-to-left shunts, Tetralogy of Fallot with pulmonary atresia and tricuspid atresia have also been reported. Because no single embryological mechanism explains all these cardiac anomalies in AOS, altered fetal hemodynamics has been proposed as a contributor [8,51,59].

### 4.4. Brain Anomalies

Neurologic anomalies are also common in AOS. A broad spectrum of intracranial anomalies has been reported, varying in severity from milder findings like idiopathic intracranial calcifications to more significant malformations such as cortical dysplasia, polymicrogyria or acrania. Additional neurological features include: seizures, developmental delays, microcephaly, spasticity, ataxia, and intellectual disabilities [8,51,59].

In this regard, Badiu Tișa et al. et al. reported a slight delay in neurological development in a 2-year-old boy diagnosed with type 2 AOS, with the psychomotor development corresponding to the age of 18 months [62].

### 4.5. Nutritional Issues

Current evidence, despite being limited, indicates that AOS may impact nutritional status. For instance, Dehdashtian et al. presented the case of a patient with AOS without major organ anomalies that may alter the normal lifespan but with poor feeding and poor weight gain [13]. Similarly, Badiu Tișa et al. reported the case of a patient without major visceral abnormalities, but with insufficient weight gain, the patient being consistently on the 3rd percentile on the weight-for-age chart. The 3rd percentile is the usual value used as the lower threshold of normal growth. A child on the 3rd percentile may be nutritionally healthy, but requires careful medical and nutritional monitoring [62,63].

An impaired nutritional status in AOS may be determined by various causes, depending on the phenotypic variability of the syndrome. In some cases, AOS is accompanied by neurological abnormalities, including developmental delays or intellectual disability, which can affect feeding ability [26,35]. Likewise, AOS can be associated with heart defects, which may further correlate with growth restriction due to factors such as fatigue during feeding or increased energy requirements [29,64].

### 4.6. Other Anomalies

Less common complications of AOS include gastrointestinal, ocular, and genital abnormalities. The gastrointestinal abnormalities include: duodenal stenosis, esophageal atresia, malrotation, obstruction, and hepatoportal disease. Ocular features include: retinal detachment, cataracts, and glaucoma. Genital malformations, although uncommon, include: cryptorchidism, inguinal hernias, and hypertrophic labia minora [8,51,59].

## 5. Prognosis in Adams–Oliver Syndrome

The prognosis in AOS is highly variable and largely dependent on the presence and severity of systemic involvement. AOS with minimal phenotypic expression does not represent a life-threatening condition. Most cases without severe visceral involvement have a favorable clinical course, and long-term survival is comparable to that of the general population. Life expectancy primarily depends on the severity of vertex aplasia and the presence of associated cardiac malformations. Superficial aplasia cutis lesions typically heal without major sequelae when properly managed. However, individuals with severe scalp and skull defects may develop complications such as hemorrhage and meningitis, which can lead to long-term morbidity and disability, and even death [13,53,65].

Likewise, severe congenital cardiac anomalies are associated with an increased mortality risk and have been reported particularly in individuals carrying *NOTCH1* pathogenic variants. In their study, Hassed et al. reviewed clinical data on 398 individuals with AOS and found that congenital heart defects and central nervous system anomalies each occurred in approximately 23% of these patients. In the same study, there were 25 deaths reported and hemorrhage, generally from ulcerated scalp defects or from intracranial vascular anomalies, was the reported cause of death for 20% of the fatal cases [53]. Therefore, early identification of symptoms that may indicate cardiac decompensation, but also systemic infection or neurological involvement, contributes significantly to improving the overall prognosis [25,28,29,66].

## 6. A Multidisciplinary Framework for the Comprehensive Management of Adams–Oliver Syndrome

AOS is mainly managed through symptom-based approaches tailored to the congenital abnormalities present in the patient. Currently, the management is multidisciplinary and usually includes physicians and nurses specialized in genetics, pediatrics, cardiology, dermatology, surgery, orthopedics, neurology, and ophthalmology. As this review will further highlight, the integration of a dietitian into the multidisciplinary team is also important for the appropriate management of AOS [11].

Overall, a coordinated, multidisciplinary approach is essential for the optimal management of AOS. The roles of the key specialists involved in the comprehensive management of AOS are outlined in Table 3.

Furthermore, it is important to note that, to date, no formal international, evidence-based clinical practice guidelines for AOS have been established. This is a common challenge for rare diseases due to the limited number of patients and the difficulty in conducting large-scale clinical trials [67]. Therefore, the current management strategies are largely based on case reports and extrapolation from recommendations for individual clinical features (e.g., management of aplasia cutis congenita or specific cardiac defects) [8,68,69].

**Table 3:** Medical specialties with key roles in the management of Adams–Oliver syndrome [8,15,26,60,70,71,72,73,74].

Medical Specialty	Role in AOS Management
Medical Genetics	Confirmatory diagnosis through genetic testing. Determine inheritance pattern. Provide genetic counseling for the family regarding recurrence risk.
Pediatrics	Central coordination of care. Monitoring of overall health, growth, and development.
Cardiology	Systematic screening at diagnosis with echocardiography for congenital heart defects, especially in cases with *NOTCH1* mutations. Long-term follow-up for cardiac anomalies.
Dermatology	Management of aplasia cutis congenita. Conservative wound care (dressings, infection prevention). Surgical consultation for large or persistent scalp defects.
Orthopedics	Assessment and management of terminal transverse limb defects.
Neurology	Evaluation for central nervous system anomalies, especially in *DOCK6*-related AOS. Management of seizures and monitoring of psychomotor development.
Ophthalmology	Screening for ocular anomalies, especially in *DOCK6*-related AOS. Management of visual deficits.
Nutrition and Dietetics	Early nutritional screening and assessment. Continuous nutritional monitoring. Management of feeding difficulties. Ensuring adequate nutrient intake to support growth and development.
Specialized Nursing	Advanced wound care for aplasia cutis congenita. Pre- and post-operative management for surgical interventions. Proactive pain assessment and management. Collaboration with rehabilitation and occupational therapy to implement functional goals and adaptive strategies. Management of prostheses/orthoses and monitoring of skin integrity. Ensuring a safe transition to the home environment with ongoing care. Comprehensive family education on all aspects of care. Providing psychosocial support and connection to advocacy groups.

AOS, Adams–Oliver syndrome; DOCK6, dedicator of cytokinesis 6.

### 6.1. Genetic Counseling

When it comes to AOS, genetic counseling is critical. The genes that are affected influence the pattern of transmission. The majority of cases show an autosomal dominant transmission, being linked to mutations in *ARHGAP31*, *DLL4*, *RBPJ*, and *NOTCH1* genes, whereas some that result from mutations in *EOGT* and *DOCK6* genes, follow an autosomal recessive pattern of transmission. Data on the frequency and distribution of mutations in large cohorts are currently limited. However, recent data suggests that autosomal recessive forms of AOS are due to biallelic mutations in *DOCK6* (60%) followed by *EOGT* (40%) [55,70,75].

Couples who are at risk should have access to genetic counseling, with the understanding that every pregnancy carries a 25% risk of having an affected offspring when the transmission is autosomal recessive and a 50% risk when the transmission is autosomal dominant, respectively [70].

### 6.2. Early Diagnosis

The early diagnosis of AOS is crucial. It improves disease outcomes by delivering care as early as possible and is, therefore, an essential public health approach in all settings [57]. Regarding antenatal diagnosis, the ultrasound examination generally reveals fetal growth restriction as well as severe reduction anomalies at the level of all limbs. When distal limb abnormalities and vertex defects are observed on ultrasounds, antenatal diagnosis can be made. When a pathogenic variant is found in a family, antenatal diagnosis may be achievable depending on the severity of the malformations in that family. In families where there is a known Adams–Oliver syndrome variant, antenatal testing, which is based on DNA analysis of amniocentesis and chorionic villus sampling, may be helpful to corroborate findings from ultrasonography and echocardiography [76].

Fetoscopy, which is typically performed under local anesthetic, is currently a low-risk procedure for the mother. Assessing the degree of expansion of skin abnormalities is one useful application for it. The scalp and limb deformities can be observed during the fetoscopy. Additionally, physicians may detect the presence of marmorata cutis. Likewise, a negative prognosis can be established with the use of the fetoscopy. The procedure helps parents by offering them a better understanding of the severity of the defects and assisting them in making decisions. The fetoscopic direct visualization can be a useful tool for differential diagnosis of rare anomalies, especially those that impact the external features of the limbs, face, genitalia, and rest of the skin, like the amniotic band (membranes) syndrome [76].

### 6.3. Interventions for Aplasia Cutis Congenita Treatment

A systematic approach is required in the management of aplasia cutis congenita. Upon birth, the lesions must be assessed for size, depth, and the presence of underlying bone defects [77]. For small, superficial defects (<2–3 cm), conservative management with wound care may suffice. This involves gentle cleansing with sterile saline and the application of dressings [78]. The choice of dressing is guided by the wound’s characteristics. For small, superficial defects, a non-adherent dressing like petrolatum gauze is sufficient, while larger or deeper lesions may benefit from hydrocolloid or foam dressings that maintain a moist wound environment, which has been shown to promote healing [77,79,80].

However, for larger defects, or those with exposed dura mater or bone, early surgical intervention is often necessary [35,65,81]. One primary goal of surgical intervention for aplasia cutis congenita in AOS is to achieve stable, durable wound closure. Likewise, the aim of surgical management for aplasia cutis congenita is to prevent life-threatening complications such as hemorrhage from the sagittal sinus or meningitis, and preserve brain function. For small, elliptical defects, simple primary closure may be possible after undermining the surrounding scalp tissue. This is the simplest approach but is limited by the size of the defect. For larger defects where primary closure is not feasible, split-thickness or full-thickness skin grafting is a common technique. While providing coverage, skin grafts on the scalp can result in alopecia and may be less durable than native scalp tissue. In some cases, allografts or xenografts may be used as a temporary biological dressing to protect the underlying structures while awaiting definitive closure [72,81,82].

In cases where the dura mater is absent or compromised, neurosurgical intervention is critical. Repair is often achieved using an artificial dura mater substitute (e.g., processed bovine pericardium, synthetic polymers) or by creating a vascularized flap from the patient’s own pericranium. This step is essential to isolate the brain from the external environment and prevent cerebrospinal fluid leakage [11,81].

Surgical options in aplasia cutis congenita management also include local and regional flaps and tissue expansion, respectively. For full-thickness scalp defects, rotating or transposing local flaps of adjacent scalp tissue can provide durable, hair-bearing coverage. This is often the preferred method for achieving the best cosmetic and functional outcome. As concerns tissue expansion, in a staged approach, tissue expanders can be placed under the adjacent healthy scalp. Over several weeks, the expanders are gradually inflated, stretching the scalp to create excess tissue that can then be advanced to cover the defect. This technique allows for the closure of very large defects with hair-bearing scalp tissue [81,83].

In addition to these strategies, the topical application of recombinant growth factors has emerged as an advanced pharmacological approach to actively stimulate and accelerate the healing process. These therapies are particularly valuable for large or slow-healing defects where conservative measures alone may be insufficient. Key growth factors used in this context include platelet-derived growth factor (PDGF), epidermal growth factor (EGF), and fibroblast growth factor (FGF). These growth factors work by mimicking the body’s natural repair mechanisms, promoting cell migration, proliferation, and differentiation to accelerate wound closure and improve the quality of the resulting scar tissue. In particular, the PDGF becaplermin (Regranex^®^) is an FDA-approved topical gel that has been shown to significantly enhance chronic wound healing. It promotes the recruitment and proliferation of fibroblasts and smooth muscle cells. Studies have demonstrated that PDGF can increase healing rates by as much as 39% compared to placebo gels. Moreover, EGF stimulates keratinocyte proliferation and migration, which is essential for re-epithelialization. Its effectiveness has been demonstrated specifically in the context of AOS. A recent case report detailed the successful conservative treatment of a large aplasia cutis congenita defect in an infant with AOS using a recombinant human EGF gel, leading to complete healing and avoiding the need for surgery. As concerns basic FGF (bFGF), this is potent in stimulating angiogenesis and fibroblast proliferation, which are critical steps in the formation of healthy granulation tissue [57,71,84].

Nevertheless, it is important to emphasize that the applicability of certain advanced diagnostic and therapeutic interventions in AOS is highly dependent on the healthcare setting. For example, complex neurosurgical repairs require specialized pediatric centers. Similarly, technologies that may aid antenatal diagnosis, such as fetoscopy, are not universally available, particularly in low-resource settings. Therefore, management must be tailored not only to the patient’s specific phenotype but also to the available local resources and expertise [85,86].

### 6.4. Nutritional Management

Nutritional screening and assessment are essential for individuals with AOS due to potential feeding difficulties, growth challenges, and the need to address underlying medical conditions. In consequence, early and ongoing nutritional assessment is crucial for optimizing the well-being of individuals with AOS. In fact, the nutritional assessment in AOS should ideally be initiated from the time of diagnosis, to identify early nutritional issues and facilitate the rapid implementation of personalized dietary interventions that may contribute to a better patient outcome. Therefore, the involvement of a registered dietitian in the multidisciplinary team caring for the patient with Adams–Oliver syndrome is critical [64,87].

The specific nutritional needs in AOS will vary based on the individual’s presentation and other health issues. Many individuals with AOS, particularly those with significant limb malformations or head abnormalities, may face difficulties with feeding. These challenges may include problems with sucking, swallowing, or managing food intake [73,88,89].

Moreover, infants affected by AOS who present with congenital heart defects, may exhibit growth failure due to a combination of increased energy expenditure and reduced nutritional intake. Caloric needs may be increased due to the enhanced respiratory effort. Furthermore, these infants may experience significant feeding fatigue, causing them to tire easily during feeds and consume insufficient volumes [69,90,91,92]. Pain from large aplasia cutis congenita lesions can also contribute to feeding refusal and oral aversion, further complicating nutritional intake [68,93]. Likewise, wound healing of large aplasia cutis congenita defects may determine increased caloric requirements [94].

Nutritional assessment helps evaluate growth patterns and identify any nutritional issues that require intervention. A comprehensive nutritional evaluation may involve a combination of tools, such as anthropometric measurements (e.g., length or height, weight, head circumference, mid-upper arm circumference, skinfold thickness), clinical examination, food intake assessment (feeding habits), caloric and protein needs calculation, and laboratory tests. These tests can help determine the patient’s detailed nutritional status. Based on nutritional assessment, modifying feeding techniques and making dietary changes to provide the adequate intake of calorigenic nutrients, vitamins, and minerals, thereby addressing deficiencies and promoting growth, are crucial steps in developing a personalized nutritional plan [73,88,89].

In cases with increased energy demands, strategies to ensure proper energy provision may be necessary (e.g., fortifying human milk or using high-calorie formulas in infants). A key nutritional intervention may also include optimizing feeding schedules, as recommending smaller, more frequent feeds can help maximize total daily intake. Moreover, enteral feeding may be required in cases of severe feeding failure to ensure adequate nutritional support [69,90,91,92].

In addition, ensuring an adequate supply of specific amino acids and micronutrients may be an important approach for supporting both somatic growth and the complex process of wound healing for aplasia cutis congenita defects. For instance, arginine is a conditionally essential amino acid that represents a substrate for nitric oxide synthesis, which improves blood flow to the wound, and a precursor for proline, a key component of collagen. Arginine supplementation has been shown to improve protein anabolism and cellular growth. Moreover, key micronutrients for wound healing include zinc, iron, copper, vitamin C, vitamin A, and vitamin D. Zinc is a crucial cofactor for numerous enzymes involved in protein and collagen synthesis, as well as immune function. Iron is essential for oxygen transport and cellular proliferation. Copper is required for the cross-linking of collagen and elastin, which provides strength and integrity to new tissue. Vitamin C is an essential cofactor for collagen synthesis and a potent antioxidant that protects new tissue from damage. Vitamin A plays a key role in epithelial cell differentiation and immune function. Vitamin D has immunomodulatory effects that are important in the inflammatory phase of wound healing [94,95,96,97].

Furthermore, in AOS, is essential for the dietitian to provide continuous nutritional monitoring. This should include frequent anthropometric measurements to promptly identify failure to thrive and periodic assessment of feeding efficacy [98]. The dietitian subsequently adapts the feeding plan based on the patient’s specific requirements. Likewise, the dietitian works with families to develop and implement individualized strategies for eating (e.g., using modified utensils in patients with upper limb deficiencies), taking into account the specific needs and abilities of each child. By formulating feasible feeding plans and providing practical education and hands-on training to families on adequate feeding, the dietitian empowers families to become active participants in their child’s nutritional care [87,94].

### 6.5. Specialized Nursing Care

The care of patients diagnosed with AOS requires a complex, multidisciplinary approach, with nurses of various specialties playing a crucial role. Nevertheless, it is important to mention that in some countries, specialized nursing roles exist, whereas in others the nursing workforce consists only of general nurses (general medical assistants) [99].

To improve quality of life and overall prognosis, nursing care in Adams–Oliver syndrome is grounded in a holistic approach, addressing both the patient’s immediate clinical needs and the family’s educational and emotional support requirements [8,11,72,74,100].

Monitoring skin integrity is essential. Assessment of cardiac, respiratory, and neurological function is particularly important in patients with extensive cranial defects or congenital cardiac malformations. Interdisciplinary collaboration with dermatology, cardiology, neurosurgery, plastic surgery, and medical genetics is fundamental. Furthermore, nursing professionals can function as care navigators, coordinating the scheduling and integration of multiple specialist consultations, thereby reducing fragmentation of care and enhancing adherence to the comprehensive management plan. The nursing role also includes extensive family education [8,72,74,100].

#### 6.5.1. Nursing Roles in the Management of Aplasia Cutis Congenita

The role of specialist nurse in the management of aplasia cutis congenita in patients with AOS is multifaceted and central to preventing complications and promoting optimal healing. The foundation of effective wound care is a thorough and systematic assessment. The nurse is responsible for continuously monitoring the wound for any changes, ensuring that the treatment plan remains appropriate for the wound’s healing stage. Preventing infection is a primary goal, especially in large defects or those with exposed dura mater. The nurse implements strict aseptic techniques during all interactions with the wound. The nurse ensures that dressings are changed at the correct frequency to manage exudate, protect the wound, and prevent maceration of the surrounding healthy tissue [101].

Dressing changes and wound manipulation can be significant sources of procedural pain, particularly in neonates. The nurse plays a critical role in proactive pain management. This involves a dual approach: administering prescribed analgesics 30–60 min prior to the procedure and utilizing non-pharmacological strategies such as swaddling, non-nutritive sucking with sucrose, and gentle handling to minimize distress. Continuous pain assessment using validated, age-appropriate scales is essential to titrate interventions effectively [102,103].

For patients with large, full-thickness scalp defects, the nurse must maintain a high index of suspicion for life-threatening complications (e.g., hemorrhage, cerebrospinal fluid leakage, systemic infection/meningitis). Nurse must monitor for any signs of bleeding and ensure that emergency supplies are readily available. In cases with dural defects, the nurse must inspect the dressing and wound for clear, watery drainage, which could indicate a cerebrospinal fluid leak, and report it immediately. Likewise, the nurse must monitor vital signs and perform neurological checks to detect early signs of systemic infection or meningitis, such as fever, lethargy, or irritability [82].

Furthermore, the specialist nurse is integral to the perioperative care of patients with AOS undergoing surgical repair of scalp defects. This role encompasses a continuum of care from pre-operative preparation to post-operative recovery and is critical for optimizing outcomes and minimizing complications. Pre-operatively, the nurse’s responsibilities include comprehensive patient and family education regarding the planned procedure, expected outcomes, and post-operative care requirements. The nurse performs baseline neurological, cardiovascular, and respiratory assessments and collaborates with the surgical team to develop a coordinated plan of care [11,104,105,106].

Post-operatively, specialist nurse’s role is multifaceted and requires a high level of vigilance. Key responsibilities include meticulous and frequent assessment of skin grafts and surgical flaps. The nurse monitors for signs of viability, such as color and temperature, to detect any evidence of vascular compromise early. To prevent infections, strict aseptic technique is maintained during all dressing changes. The nurse monitors the wound for signs of infection (e.g., erythema, purulent drainage, fever) and administers therapy as prescribed. Moreover, the nurse manages proactively procedural and post-operative pain to ensure patient comfort, both by implementing the prescribed pharmacological treatment (e.g., analgesics) and autonomously initiating non-pharmacological interventions (e.g., swaddling, non-nutritive sucking) [102,103].

Following dural repair, the specialist nurse watches for the appearance of signs of increased intracranial pressure, seizures, or cerebrospinal fluid leakage from the wound, and reports any changes immediately to the neurosurgical team. The nurse provides ongoing emotional support to the family, offers clear updates on the patient’s progress, and provides hands-on training for any wound care that will be required after discharge [78,107,108,109,110].

#### 6.5.2. Nursing Roles in Rehabilitation and Occupational Therapy

The rehabilitation nurse plays a pivotal role in translating the therapeutic goals set by physical and occupational therapists into the patient’s daily life, ensuring a seamless continuum of care for children with AOS. This role is fundamentally collaborative, acting as a bridge between the therapy team, the family, and the patient to maximize functional independence [111,112].

A key responsibility is the management of prostheses and orthoses, which often begins as early as 6–8 months of age for upper limb deficiencies. The nurse is tasked with performing regular skin integrity assessments under these devices to prevent pressure injuries, educating the family on proper device application and maintenance, and monitoring for any signs of neurovascular compromise. Furthermore, the nurse works with the family to develop and implement creative adaptive strategies for activities of daily living, such as using adapted clothing for easier dressing, thereby empowering the child and reducing caregiver burden [111,113].

#### 6.5.3. Nursing Roles in Home Healthcare

The home health nurse is essential for ensuring a safe and effective transition from the hospital to the home, acting as the crucial link in the continuum of care for patients with AOS. A primary responsibility upon discharge is to conduct a thorough assessment of the home environment. This includes evaluating the physical space for safety and accessibility, ensuring adequate hygiene standards for performing wound care, and confirming proper storage for medical supplies and medications. In this setting, the nurse provides direct, hands-on clinical care, skillfully adapting hospital-based protocols for ongoing wound management to the practical realities of the home. This ensures that the high standard of care initiated in the hospital is maintained, reducing the risk of complications and preventing rehospitalization [114,115].

Furthermore, the home health nurse serves as the frontline professional for reinforcing education and coordinating local services. While the hospital team provides initial instruction, the home health nurse observes the family’s caregiving techniques in practice, answers practical questions that arise during daily care, and builds the family’s confidence and competence in managing complex needs. They act as the “eyes and ears” for the specialist team, monitoring the patient’s progress and reporting any subtle changes or concerns. Critically, the home health nurse connects the family with tangible, local community resources, such as local physical and occupational therapy providers, and durable medical equipment suppliers, thereby translating the broad recommendations of the multidisciplinary team into a functional, real-world support system [111,115,116].

#### 6.5.4. Nursing Roles in Family Education and Psychosocial Support

Given the rarity and complexity of AOS, nurses play a vital role in educating and supporting the family. The emotional and practical burden on caregivers is substantial [117].

Family education should focus on understanding the disease pathology, the need for periodic medical evaluations, and the principles of wound care at home. Families should be instructed to recognize warning signs (e.g., signs of infection, neurological changes) and to seek prompt medical attention to prevent the severe progression of complications. The use of the “teach-back” method, where caregivers explain the instructions in their own words, is an effective strategy to ensure comprehension and has been shown to improve health outcomes [118].

Furthermore, it is crucial to acknowledge the family’s emotional journey and connect them with vital support systems. This includes referrals to social workers, psychologists, and patient advocacy groups such as national organizations for rare disorders, which provide invaluable resources and a sense of community for families affected by rare diseases [119,120].

**Figure 1:**
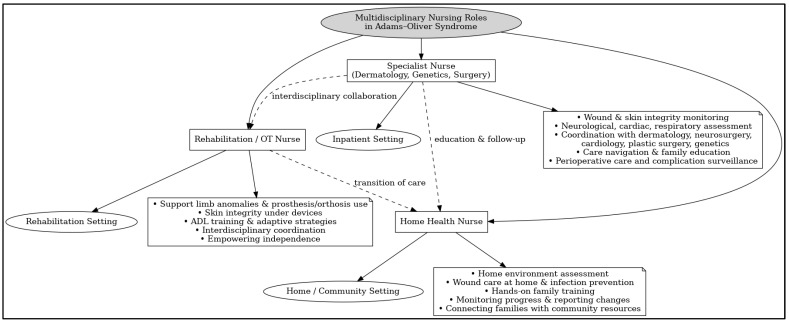
Multidisciplinary Nursing Roles in Adams–Oliver Syndrome (Abbreviations: OT, Occupational Therapy; ADL, activities of daily living).

To summarize, Figure 1 presents a flowchart illustrating the distinct and collaborative roles of nurses across different specialties (specialist nurse, rehabilitation/occupational therapy nurse, home health nurse) and settings (inpatient, rehabilitation, home/community) in the multidisciplinary management of AOS.

## 7. Conclusions

Adams–Oliver syndrome (AOS) is a clinically and genetically heterogeneous disorder, with its pathogenesis primarily rooted in defective vascular development. The molecular basis, involving mutations in six key genes (*ARHGAP31*, *RBPJ*, *NOTCH1*, *DLL4*, *DOCK6*, and *EOGT*) that disrupt critical cellular pathways including Notch signaling and Cdc42/Rac1-mediated cytoskeletal regulation, explains the wide phenotypic variability, from aplasia cutis congenita and limb defects to severe cardiovascular and neurological anomalies. The management of AOS is inherently complex and necessitates a well-coordinated, multidisciplinary team.

The present review highlights that beyond the well-documented clinical, genetic, and molecular features, nutritional status is an important but often under-recognized aspect that can significantly impact growth and development in AOS patients. Therefore, the inclusion of a registered dietitian for early and ongoing nutritional assessment and intervention is essential. Furthermore, the role of specialized nursing care is critical, not only for the complex management of skin defects and the prevention of life-threatening complications but also for providing crucial education and psychosocial support to patients and their families.

## Figures and Tables

**Table 1:** The genetic and molecular etiology of Adams–Oliver syndrome.

AOS Type	AOS Causal Gene	Inheritance Pattern	Protein Function/Signaling Pathway	References
AOS1	*ARHGAP31*	Autosomal dominant	GTPase-activating protein for Cdc42 and Rac1; cytoskeleton regulation	[17,37]
AOS3	*RBPJ*	Autosomal dominant	Transcriptional regulator; the main effector of the Notch signaling pathway	[20,38]
AOS5	*NOTCH1*	Autosomal dominant	Receptor in the Notch signaling pathway	[28,39]
AOS6	*DLL4*	Autosomal dominant	Ligand for the NOTCH1 receptor; Notch signaling pathway	[24,34]
AOS4	*EOGT*	Autosomal recessive	O-GlcNAc transferase; glycosylation of proteins in the Notch signaling pathway	[9,19]
AOS2	*DOCK6*	Autosomal recessive	Guanine nucleotide exchange factor for Cdc42 and Rac1; cytoskeleton regulation	[18,26]

AOS, Adams–Oliver syndrome; ARHGAP31, Rho GTPase Activating Protein 31; RBPJ, recombination signal binding protein for immuno-globulin kappa J region; NOTCH1, Notch intracellular domain; DLL4, Notch ligand Delta-like ligand 4; EOGT, eukaryotic growth factor domain specific O-linked N-acetylglucosamine transferase; DOCK6, dedicator of cytokinesis 6.

**Table 2:** Genotype–Phenotype Correlations in Adams–Oliver Syndrome.

AOS Type	AOS Causal Gene	Associated Clinical Features	References
AOS1	*ARHGAP31*	Classic AOS phenotype with aplasia cutis congenita and terminal transverse limb defects. Cutis marmorata telangiectatica congenita is common.	[17,37,40]
AOS3	*RBPJ*	Variable phenotype with aplasia cutis congenita and limb defects. Intellectual disability has been reported in some cases.	[20,38,53]
AOS5	*NOTCH1*	Strongly associated with congenital cardiac malformations.	[25,28,29,54]
AOS6	*DLL4*	Highly variable phenotype, from isolated aplasia cutis congenita to classic AOS. Cardiovascular anomalies can be present.	[24,34,54]
AOS4	*EOGT*	Variable phenotype, often with large aplasia cutis congenita but sometimes with milder limb defects. Lower frequency of neurological deficits compared to *DOCK6*.	[9,19,53]
AOS2	*DOCK6*	Often associated with a more severe phenotype. Strongly linked to terminal transverse limb defects. Central nervous system and ocular abnormalities are also frequently reported.	[18,33,51,55]

AOS, Adams–Oliver syndrome; ARHGAP31, Rho GTPase Activating Protein 31; RBPJ, recombination signal binding protein for immuno-globulin kappa J region; NOTCH1, Notch intracellular domain; DLL4, Notch ligand Delta-like ligand 4; EOGT, eukaryotic growth factor domain specific O-linked N-acetylglucosamine transferase; DOCK6, dedicator of cytokinesis 6.

## Data Availability

No new data were created or analyzed in this study. Data sharing is not applicable to this article.

## Abbreviations

Adams–Oliver syndrome, AOS; dedicator of cytokinesis 6, DOCK6; epidermal growth factor, EGF; eukaryotic growth factor domain specific O-linked N-acetylglucosamine transferase, EOGT; fibroblast growth factor, FGF; GTPase-activating protein, GAP; guanine nucleotide exchange factor, GEF; Mastermind-like, MAML; Neurogenic locus notch homolog protein 1, NOTCH1; Notch intracellular domain, NICD; Notch ligand Delta-like ligand 4, DLL4; platelet-derived growth factor, PDGF; recombination signal binding protein for immunoglobulin kappa J region, RBPJ; Rho GTPase Activating Protein 31, ARHGAP31.
